# Purification and Functional Characterization of a Biologically Active Full-Length Feline Immunodeficiency Virus (FIV) Pr50^Gag^

**DOI:** 10.3390/v11080689

**Published:** 2019-07-27

**Authors:** Anjana Krishnan, Vineeta N. Pillai, Akhil Chameettachal, Lizna Mohamed Ali, Fathima Nuzra Nagoor Pitchai, Saeed Tariq, Farah Mustafa, Roland Marquet, Tahir A. Rizvi

**Affiliations:** 1 Department of Microbiology & Immunology, College of Medicine and Health Sciences (CMHS), United Arab Emirates University (UAEU), Al Ain 2000, UAE; 2 Department of Anatomy, College of Medicine and Health Sciences (CMHS), United Arab Emirates University (UAEU), Al Ain 20000, UAE; 3 Department of Biochemistry, College of Medicine and Health Sciences (CMHS), United Arab Emirates University (UAEU), Al Ain 20000, UAE; 4 Université de Strasbourg, CNRS, Architecture et Réactivité de l’ARN, UPR 9002 Strasbourg, France

**Keywords:** feline immunodeficiency virus (FIV), retroviral RNA packaging, viral assembly, Pr50^Gag^ protein expression, Gag protein purification, His-tag fusion protein

## Abstract

The feline immunodeficiency virus (FIV) full-length Pr50^Gag^ precursor is a key player in the assembly of new viral particles. It is also a critical component of the efficient selection and packaging of two copies of genomic RNA (gRNA) into the newly formed virus particles from a wide pool of cellular and spliced viral RNA. To understand the molecular mechanisms involved during FIV gRNA packaging, we expressed the His_6_-tagged and untagged recombinant FIV Pr50^Gag^ protein both in eukaryotic and prokaryotic cells. The recombinant Pr50^Gag^-His_6_-tag fusion protein was purified from soluble fractions of prokaryotic cultures using immobilized metal affinity chromatography (IMAC). This purified protein was able to assemble in vitro into virus-like particles (VLPs), indicating that it preserved its ability to oligomerize/multimerize. Furthermore, VLPs formed in eukaryotic cells by the FIV full-length Pr50^Gag^ both in the presence and absence of His_6_-tag could package FIV sub-genomic RNA to similar levels, suggesting that the biological activity of the recombinant full-length Pr50^Gag^ fusion protein was retained in the presence of His_6_-tag at the carboxy terminus. Successful expression and purification of a biologically active, recombinant full-length Pr50^Gag^-His_6_-tag fusion protein will allow study of the intricate RNA-protein interactions involved during FIV gRNA encapsidation.

## 1. Introduction

Genomic RNA (gRNA) packaging during the retroviral life cycle has been a topic of intense investigation over the last few decades. It has become increasingly clear that during this process, two copies of “full-length” unspliced gRNA are preferentially/selectively packaged in a dimeric form into the nascent virus particles over the viral spliced, as well as cellular RNA present in the infected cell [1,2,3,4,5,6,7]. Sequences responsible for selective packaging of unspliced gRNA are called “packaging signal/packaging determinants” or “psi” (ψ) and have been found to be universally present at the 5′ end of all retroviral genomes [1,2,3,4,5,6,8]. Interestingly, without any exception, all retroviral ψ sequences have been shown to assume higher order structures containing several physical motifs in the form of stem loops [1,2,3,4,5,6,7]. During the course of retroviral gRNA packaging, specific interactions must take place between psi RNA and full-length Gag [9,10,11,12]. However, not much is known about how the Gag precursor polypeptide achieves this selective gRNA packaging, primarily due to the lack of availability of a purified full-length Gag precursor protein. We and others have tried to narrow that gap by purifying these full-length Gag proteins for three different retroviruses that show different assembly/maturation processes: human immunodeficiency virus type 1 (HIV-1; [9,13]), a type C lentivirus that assembles at the plasma membrane, and the Mason–Pfizer monkey virus (MPMV; [14]) and mouse mammary tumor virus (MMTV; [15]), retroviruses that assemble intracellularly [16].

The availability of full-length Gag proteins could lead to a better understanding of gRNA encapsidation by retroviruses since it is the natural partner with whom the viral genomes initially interact during this process. In the case of HIV-1, earlier experiments studying gRNA packaging led to erroneous results due to the lack of availability of the HIV-1 full-length Gag. These experiments were conducted using different domains of Gag, which suggested that the HIV-1 packaging sequences primarily reside in the third stem loop (SL3) of the viral packaging signal, since it contained a tetra loop (GGAG), which was shown to bind with high affinity to the nucleocapsid (NC) domain of HIV-1 Gag [17,18]. However, with the availability of HIV-1 full-length Gag precursor (Pr55^Gag^), it has been shown that instead of SL3, a purine-rich internal loop (G//AGG) in SL1 in the packaging determinants of HIV-1 functions as the primary Pr55^Gag^ binding site to facilitate HIV-1 gRNA packaging [9,10,11,12,19]. Such contradictory observations suggest that the selective retroviral gRNA packaging from a milieu of viral spliced and cellular RNAs is a multifaceted process that must be studied in the context of the full-length Gag precursor polypeptide. This conclusion is reinforced by the recent observation that the C-terminal p6 domain of HIV-1 Pr55^Gag^ is crucial for specific binding to gRNA [20]. Consequently, there is a gap in the proper understanding of the intricacies involved in the selective retroviral gRNA packaging process, attributed to the lack of availability of a biologically active full-length Gag precursor polypeptide for most retroviruses.

Feline immunodeficiency virus (FIV) is a lentivirus that has been shown to infect a number of different species of cats, in whom it causes a progressive immune dysfunction that is similar to HIV-1 induced acquired immunodeficiency syndrome (AIDS) in humans [21,22,23,24,25,26]. Owing to its many close parallels with HIV-1 in replication and pathobiology, and because it is the only non-primate model that induces AIDS in its natural host, FIV has positioned itself as an important animal model to develop and test anti-AIDS vaccines and therapies [21,27,28,29,30,31]. FIV, being a feline retrovirus, is phylogenetically distant from human and primate retroviruses, thus making it a potentially ideal vector of choice for human gene therapy. Furthermore, being a lentivirus, FIV has the ability to infect and transduce non-dividing cells, making FIV-based vectors a hotly pursued non-human/non-primate retroviral vector system for human gene therapy [21,31,32,33,34,35]. From these perspectives, delineating the molecular mechanisms of the FIV life cycle, especially how the FIV full-length Gag precursor (Pr50^Gag^) specifically packages the viral gRNA from a variety of cellular and viral spliced RNAs is crucial for the development of efficient and safe FIV-based vectors for human gene therapy.

Although studied extensively, little is known about the molecular mechanisms of FIV gRNA packaging. A series of studies have identified sequences of the FIV genome crucial in augmenting gRNA packaging [21]. Located at the 5’ end of the viral genome similar to other retroviruses, they extend into the Gag coding sequences [36,37,38,39,40,41]. Furthermore, the FIV packaging signal RNA is a highly structured motif that is bipartite in nature, consisting of two discontinuous core regions. The intervening sequences between these two regions are nonessential and can be deleted/substituted without disrupting gRNA packaging efficiency [42,43,44,45].

The dimerization of retroviral gRNA is intrinsically related to RNA packaging, as it is a prerequisite for RNA encapsidation [3,7,46,47]. The selective 2’-hydroxyl acylation analyzed by primer extension (SHAPE) analysis of the 5’ end of the FIV genome involved in these processes has shown this region to fold into five stem loops (SL1-5), held together by long range interactions (LRI) [42,44,45]. A palindromic sequence (pal; 5’AAUGGCCAUU3’) within SL5 present in *gag* is thought to function as the dimerization initiation site (DIS) for FIV gRNA [42,44,45]. Although four of the stem loops (SL1-4) within this region are static in nature, the FIV LRI has been shown to adopt an alternative, yet similarly-conserved, RNA conformation, in which the putative SL5 DIS is occluded, and which may favor translation and splicing over encapsidation [42]. Since FIV gRNA dimerization is initiated at a site within the Gag coding region, such a scenario is likely to confer high specificity to packaging, limited exclusively to the unspliced viral RNA. Moreover, the DIS is a purine-rich sequence and the presence of these purines in the packaging sequences of retroviral gRNA has been proposed to facilitate RNA encapsidation by functioning as a potential Gag binding site [9,47,48,49,50,51]. Therefore, it is reasonable to hypothesize that in the case of FIV, the palindromic sequence within SL5, in addition to functioning as DIS, could also be involved in RNA-protein interactions during genomic RNA encapsidation by functioning as a potential Gag binding site.

The *gag* gene of FIV encoding for the precursor polypeptide, Pr50^Gag^, is processed by the viral protease into its constituent mature proteins: NH_2_-p15 (matrix; MA), p24 (capsid; CA), p1 (spacer peptide), p13 (nucleocapsid; NC), p2 (spacer peptide)-COOH [26,52]. Like all retroviruses, FIV Pr50^Gag^ assembles to form an immature capsid and expression of the *gag* gene alone results in the assembly and release of immature virus-like particles (VLPs) [21,22]. During the assembly of FIV particles, the viral Pr50^Gag^ protein must specifically select the viral gRNA from a variety of cellular and viral spliced RNAs. However, how Pr50^Gag^ selectively chooses gRNA for packaging remains unclear. For example, it is not yet fully understood whether the sorting mechanism between gRNA and spliced RNA is only dependent on the inherent binding of the full-length Gag precursor (Pr50^Gag^) or whether there are other steps in the FIV life cycle also responsible in selecting gRNA, such as nucleocytoplasmic export and cellular compartmentalization of gRNA, as has recently been advocated for in HIV-1 [50,53,54,55,56,57,58]. Therefore, to understand the molecular intricacies involved in FIV gRNA packaging, we must express and purify Pr50^Gag^ and understand its biological, biochemical, and biophysical attributes.

Only one study in the literature has reported the expression and purification of FIV Pr50^Gag^ from *Escherichia coli* (*E. coli*) [59]. However, its appropriateness for in vitro RNA binding assays, such as band-shift assays and footprinting experiments, has not been established. Therefore, in our attempt to identify Gag binding site(s) on FIV gRNA, as a first step, we sub-optimally over-expressed recombinant full-length Pr50^Gag^-His_6_-tag protein in *E. coli* and purified large amounts from bacterial soluble fractions employing immobilized metal affinity chromatography (IMAC) and high-pressure liquid chromatography (HPLC), as has recently been described for HIV-1, MPMV, and MMTV [13,14,15,60,61]. The purified recombinant full-length Pr50^Gag^-His_6_-tag protein showed an intrinsic ability to assemble into virus-like particles in eukaryotic cells. Furthermore, VLPs formed by the recombinant full-length Pr50^Gag^-His_6_-tag protein in the eukaryotic cells could package the FIV gRNA, further confirming the biological activity of the purified recombinant protein. The availability of biologically active recombinant full-length Pr50^Gag^-His_6_-tag protein should pave the way to further delineate the molecular mechanism(s) during selective FIV gRNA packaging process by establishing the differential binding abilities of the protein to different structural motif(s) on the full-length unspliced gRNA.

## 2. Materials and Methods

### 2.1. FIV Strain and Nucleotide Designations

The FIV_Petaluma_ (34TF10) strain was used for this study, and the nucleotide numbers refer to the GenBank accession number M25381.1 [62].

### 2.2. Prokaryotic FIV Gag Expression Plasmid Construction

The FIV full-length *gag* sequences from nucleotides 628 to 1980 without codon optimization were commercially synthesized (Macrogen, Seoul, South Korea) with flanking *Nco*I and *Xho*I sites at the 5’ and 3’ ends respectively, to enable cloning into the prokaryotic expression vector pET28b (+) [9,13]. For the ease of cloning, the sequence CTCGAG corresponding to two *Xho*I sites in the native FIV Gag sequence was modified to CTAGAG (at nucleotide position 1408) and CACGAG (at the nucleotide position 1451), respectively, during gene synthesis to eliminate the *Xho*I sites while retaining the amino acid sequence. The synthesized FIV full-length *gag* was cleaved with *Nco*I and *Xho*I and cloned into the *Nco*I and *Xho*I sites of the pET28b (+). Such a cloning strategy kept the *gag* sequences in frame with a His_6_-tag at the C-terminus, resulting in the prokaryotic expression plasmid (VP10), which should express recombinant full-length Pr50^Gag^ His_6_-tag fusion protein with a predicted molecular weight of 50.3 kilodaltons (kDa) as computed by ExPASy- pI/Mw tool. The VP10 clone was sequenced to ensure that there were no point mutations introduced during gene synthesis. 

### 2.3. Eukaryotic FIV Gag Expression Plasmid Construction

A pcDNA3 (Thermo Fischer Scientific, Waltham, MA, USA)-based eukaryotic expression vector expressing the recombinant full-length FIV Gag (Pr50^Gag^-His_6_-tag) was created by amplifying the FIV *gag* gene from VP10 using primers OTR1384 and OTR1385. OTR1384 (5’**CCG** *CTC GAG* GCC GCC ACC ATG GGG AAT GGA CAG GGG CG 3’; nucleotides 628–647) is the forward primer containing 3 non-viral nucleotides (bold) followed by an *Xho*I site (italized) and a Kozak sequence (underlined) upstream of *gag* sequences. OTR1385 (5’CCG *CTC GAG* **TTA** gtg gtg gtg gtg gtg gtg TAA ATC CAA TAG TTT CTC CTC CAT T 3’; nucleotides 1977–1953) is the reverse primer containing 3 non-viral nucleotides (underlined) followed by an *Xho*I site (italicized), termination codon (bold), and His_6_-tag sequence (lower case) downstream of the *gag* sequences. Similarly, a full-length FIV Gag without His_6_-tag was created by amplifying the FIV *gag* gene from VP10 using primers OTR1384 and OTR1386. OTR1386 is the reverse primer (5’CCG *CTC GAG* **TTA** TAA ATC CAA TAG TTT CTC CTC CAT T 3’; nucleotides 1977–1953) containing 3 non-viral nucleotides (underlined), followed by an *Xho*I site (italicized) and a termination codon (bold) downstream of Gag. Amplifications were setup using the Phusion High-Fidelity PCR Kit (New England Biolabs, Ipswich, MA, USA), and standard PCR conditions were followed: initial denaturation at 98 °C for 1 min, followed by 30 cycles of denaturation at 98 °C for 30 s, annealing at 61 °C for 30 s, extension at 72 °C for 30 s, and a final extension at 72 °C for 10 min. PCR amplified products with His_6_-tag (amplicon size 1379 base pairs) and without His_6_-tag (amplicon size 1361 base pairs) were digested with *Xho*I and cloned into the *Xho*I sites of the pcDNA3-a eukaryotic expression vector to generate AD2 and AD3, respectively. For the efficient nuclear export of Gag mRNA, the MPMV constitutive transport element (CTE; [63]) was cloned downstream of the *gag* stop codon, as described previously [14,15]. Both AD2 and AD3 clones were confirmed by sequencing.

### 2.4. Bacterial Strains and Culture Media for Cloning and Expressing Full-Length Recombinant FIV Pr50^Gag^-His_6_-Tag Protein

The ligated mixtures during the cloning process were transformed into the DH5α strain of *E. coli* using Luria-Bertani (LB) media in the presence of 50 µg/mL of kanamycin. For bacterial protein expression, VP10 was transformed into the BL21(DE3) strain of *E. coli* and cultured in an LB medium containing kanamycin. Analytical preparations of full-length recombinant Pr50^Gag^ His_6_-tag fusion protein were prepared as described previously [14,15].

### 2.5. Eukaryotic Expression of Recombinant Full-Length FIV Pr50^Gag^ Protein

Expression of full-length FIV Pr50^Gag^ both with and without His_6_-tag (AD2 and AD3 respectively) was tested in transient transfections of human embryonic kidney (HEK) 293T cells using a calcium phosphate kit, as described previously [14,15]. Briefly, transfections were performed in triplicate in 6-well plates using 4 µg of full-length FIV Gag eukaryotic expression plasmids (AD2 and AD3) along with 2 µg of an FIV-based transfer vector, MB87 [36]. For measuring transfection efficiency, a secreted alkaline phosphatase expression plasmid (pSEAP) was used at a concentration of 100 ng per well. The supernatants from the transfected cultures were harvested roughly 60 h post transfection and cellular debris was cleared by low speed centrifugation. The VLPs were pelleted by subjecting the clarified supernatant to ultracentrifugation at 70,000× *g* with a 20% (*w*/*v*) sucrose cushion. The pelleted VLPs were resuspended in a TNE buffer (50 mM Tris-HCl, pH 7.4; 100 mM NaCl; 1 mM EDTA pH 8.0). Cellular and viral RNAs were extracted using TRIzol, following the manufacturer’s recommendations.

### 2.6. Transmission Electron Microscopy (TEM) of Eukaryotically-Expressed Virus-Like Particles (VLPs)

VLP formation by the recombinant FIV Pr50^Gag^ protein with and without His_6_-tag (AD2 and AD3, respectively) following transfection in 293T cells was observed using transmission electron microscopy (TEM; FEI Tecnai Biotwin Spirit G2). Briefly, ~60 h following transfection, cells were harvested, pelleted, and washed with 0.1 M phosphate buffered saline (PBS). The fixation, ultrathin (95 nm) sectioning of the embedded samples and staining for TEM was performed as described previously [14,15].

### 2.7. Evaluation of RNA Packaging by FIV Gag Virus-Like Particles (VLPs) Using Reverse Transcriptase PCR (RT-PCR) and qPCR Using SYBR Green

Packaging of FIV RNA into the VLPs was monitored by RT-PCR, as described previously [14,15], but with modifications. Briefly, RNA preparations were subjected to DNase treatment to eliminate any contaminating DNA that may have been carried forward from the transfected cultures. Following DNase treatment, amplification on both cytoplasmic and viral RNA preparations were performed using transfer vector (MB87) specific primers, OTR660 (5’ GAG GAC TTT TGA GTT CTC CCT TGA GGC 3’; nucleotides 230–256) and OTR662 (5’ AGC AGG AGT TCT GCT TAA CAG CTT TC 3’; nucleotides 440–415, amplifying a 210 base pair region between U5 and UTR), to ensure that the RNA preparation were not contaminated with any plasmid DNA. After ensuring that RNA preparations were devoid of any contaminating plasmid or genomic DNA by PCR, they were converted into cDNA using random hexamers (5’NNNNNN3’) and MMLV reverse transcriptase (Promega, Madison, WI, USA). Finally, cDNAs were amplified with the same vector-specific primers as described above (amplicon size 210 base pairs) to assess the ability of Pr50^Gag^ VLPs to package transfer vector (MB87) RNA. Amplifications were setup using PCR master mix (Promega, USA) and standard PCR conditions were followed: initial denaturation at 94 °C for 2 min, followed by 30 cycles of denaturation at 94 °C for 45 s, annealing at 65 °C for 45 s, and extension at 72 °C for 45 s. An in-house SYBR Green-based qPCR assay was developed to quantitate the relative packaging of the FIV transfer vector (MB87) RNA by VLPs using the same primer set (OTR660/OTR662; 400 nM) and Solis BioDyne 5× Hot FirePol EvaGreen qPCR Supermix (Solis BioDyne, Tartu, Estonia). Expression of the FIV-specific signal was normalized to the endogenous β-actin expression by using spliced β-actin-specific primers [64] at a concentration of 100 nM each using the 2^−ΔΔCT^ method. Appropriate primer concentrations for the assay were determined empirically by conducting test assays, and the primer concentration that gave a single, narrow peak upon melt curve analysis with the maximum amplification was chosen. To circumvent any non-specific signal, the annealing and extensions were performed at 72 °C for 40 cycles using the ABI QuantStudio 7 or 3 machines (Applied Biosystems, Foster City, CA, USA)

### 2.8. Expression of Recombinant FIV Pr50^Gag^-His_6_-tag Protein in E. coli

Large scale expression of the recombinant Pr50^Gag^-His_6_-tag protein was initiated using a starter culture with kanamycin (50 µg/mL), which was inoculated with a single colony of bacteria (BL21 (DE3)) and grown overnight at 37 °C. The following day, this starter culture was used to inoculate 500 ml LB media in 2 liter baffled flasks containing kanamycin (50 μg/mL) and 1% glucose, as described previously [14,15]. The cultures were allowed to grow to an OD_600_ of 0.6, and then cultures were induced with isopropyl β-d-1-thiogalactopyranoside (IPTG), as described previously [14,15]. The induced cultures were grown for another 4 h at 28 °C and then pelleted by centrifugation at 6300× *g* for 15 min at 4 °C. The bacterial pellets were stored at −80 °C until further use.

### 2.9. Affinity Purification and Size Exclusion Chromatography of Recombinant FIV Pr50^Gag^-His_6_-Tag Protein

The recombinant Pr50^Gag^-His_6_-tagged protein was purified based on the principle of IMAC, as described previously [14,15]. Briefly, the frozen bacterial pellets were lysed using the CelLytic B buffer supplemented with benzonase, lysozyme, and EDTA-free protease inhibitor. Following lysis, the protein was purified on a Ni^2+^-immobilized HisTrap fast flow (FF) 5 mL column. An Amicon Ultra 15 device with a 30 kDa cut-off was used for concentrating the eluted protein. Coomassie Brilliant Blue staining and western blotting of the concentrated fractions were performed to establish the quality of the protein. Finally, the concentrated protein was loaded onto a Superdex 200 increase 10/300 GL column for further purification to homogeneity. Fractions that gave a peak were collected and then analyzed by sodium dodecyl sulfate-polyacrylamide gel electrophoresis (SDS-PAGE). The absorbance ratio at 260 and 280 nm ensured the purity of the protein after size exclusion chromatography. Finally, aliquots of purified Pr50^Gag^-His_6_-tag fusion protein were pooled and stored at −80 °C.

### 2.10. Sodium Dodecyl Sulfate-Polyacrylamide Gel Electrophoresis (SDS-PAGE) and Western Blotting

The Pr50^Gag^-His_6_-tagged protein was monitored for expression and purification using SDS-PAGE and western blotting. Protein samples were run on a 4%–12% ExpressPlus^TM^ PAGE gel, electrophoresed under reducing conditions, and stained using Coomassie Brilliant Blue, as described previously [14,15]. For western blot analysis, duplicate gels were transferred onto nitrocellulose membranes and probed with an FIV α-p24 monoclonal antibody (Biodesign International, Saco, Maine, USA) and an α-His_6_ monoclonal antibody-horseradish peroxidase (HRP) conjugate, as described previously [14,15].

### 2.11. In Vitro Assembly to Form Virus-Like Particles (VLPs) by the Recombinant Full-Length Pr50^Gag^-His_6_-Tag Fusion Protein Expressed in E. coli

In vitro assembly of VLPs was performed as described recently for MPMV and MMTV full-length Gag [14,15]. Briefly, the purified recombinant Pr50^Gag^-His_6_-tagged protein expressed in bacteria was resuspended in an assembly buffer (50 mM Tris (pH 7.4), 1.0 M NaCl) at a concentration of 2 mg/mL. Next, protein in the assembly buffer was mixed with yeast tRNA at a nucleic acid to protein ratio of 4% (*w*/*w*) and placed in a Slide-A-Lyzer® 10 K dialysis cassette G2 (Thermo Scientific). The dialysis cassette containing the mixture of protein and yeast tRNA was dialyzed against 20 mM Tris (pH 7.4) containing 150 mM NaCl and 10 mM dithiothreitol (DTT) overnight at 4 °C. Following dialysis, 8–10 µL of protein and yeast tRNA was spotted on a carbon coated formvar grid (Proscitech, Queensland, Australia), air dried, and stained with 1% uranyl acetate for observation using TEM.

## 3. Results and Discussion

### 3.1. Full-Length FIV Pr50^Gag^ Protein, Both with and without a His_6_-Tag Can Be Expressed in Eukaryotic Cells, Capable of Making Virus-Like Particles (VLPs) That Can Package FIV gRNA

**Figure 1:**
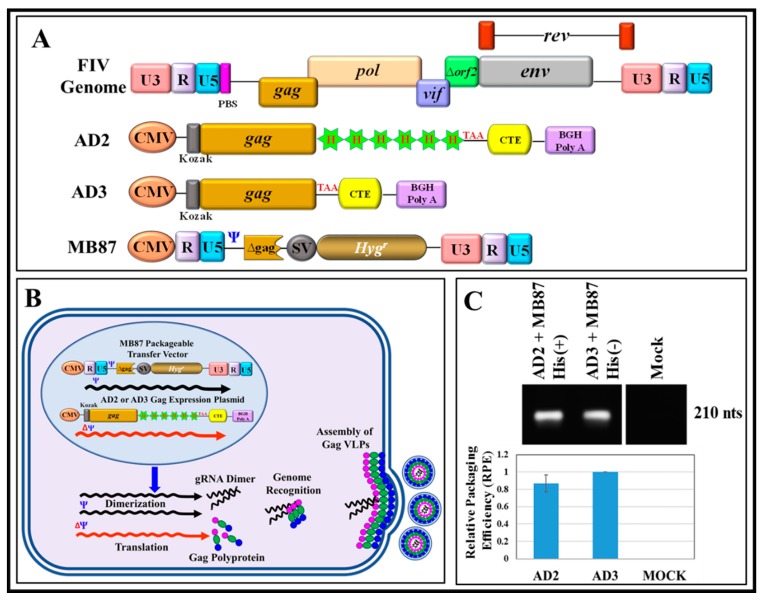
In vivo genetic complementation assay to assess the expression of a full-length feline immunodeficiency virus (FIV) Pr50^Gag^ protein, both with and without His_6_-tag, resulting in the release of virus-like particles (VLPs) that can package FIV sub-genomic RNA. (**A**) Schematic representation of the FIV genome, FIV full-length eukaryotic Gag expression plasmids with His_6_-tag (AD2) and without His_6_-tag (AD3), and the FIV sub-genomic transfer vector, MB87 [36] serving as a source of packageable RNA. (**B**) Graphical representation of the design of the genetic complementation assay. Following co-transfection of the transfer vector MB87 with either of the Gag-expression vectors (AD2 and AD3) in 293T cells should result in production of VLPs containing MB87-specific RNA due to the presence of the packaging signal (Ψ) on the vector. (**C**-upper panel) RT-PCR amplification of MB87, an FIV-based transfer vector RNA, packaged by VLPs from His_6_-tag (+) and His_6_-tag (−) Gag clones from a representative experiment. (**C**-lower panel) The relative packaging efficiency (RPE) of the FIV-based transfer vector (MB87) RNA by VLPs produced by Gag expression plasmids either with His_6_-tag (AD2) or without His_6_-tag (AD3), as measured by quantitative real time RT-PCR (qRT-PCR) conducted in triplicates.

**Figure 2:**
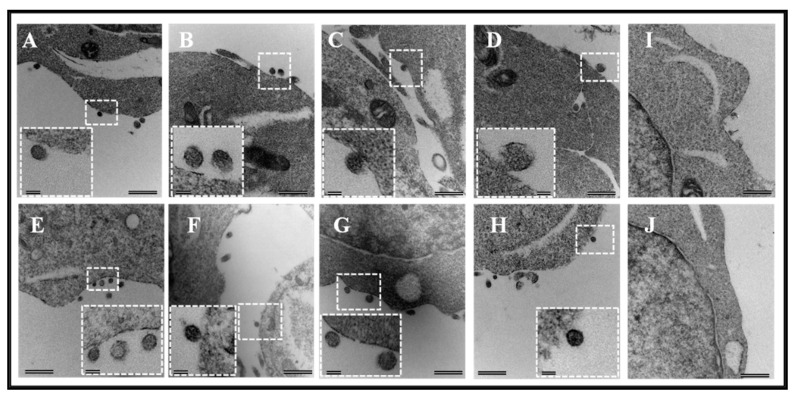
Assembly and budding of virus-like particles (VLPs) by full-length FIV Pr50^Gag^ in eukaryotic cells. The transfected 293T cells (~ 60 hours following transfection) were fixed for electron microscopy. Characteristic images of VLPs resembling immature virions budding as well as intermediates associated with the plasma membrane are shown when AD2 (full-length FIV Pr50^Gag^ expression with His_6_-tag) (**A**–**D**) and AD3 (full-length FIV Pr50^Gag^ expression without His_6_-tag) (**E**–**H**) were transfected into 293T cells. No budding of VLPs was observed when the cells were transfected with the eukaryotic expression vector alone, pcDNA3, as a negative control (**I**,**J**). Areas from where electron micrographs were taken at higher magnification are demarcated as dashed boxes and shown within the pictures as insets. Scale bars represent 500 nm (26,500× magnification) for the wide-field pictures and 100 nm (87,000× magnification) for the insets, respectively.

In this study, our goal was to express and purify a full-length FIV Pr50^Gag^-His_6_-tag fusion protein that could be used for RNA-protein interaction studies, to help establish the role of FIV Gag in gRNA packaging. However, there has been some concern that the presence of a positively-charged His-tag may favor interactions with the nucleic acids on its own [61]. Therefore, as a first step, we cloned the FIV *gag* (Pr50^Gag^) both in the presence (AD2; Figure 1A) and absence (AD3; Figure 1A) of the His_6_-tag in a eukaryotic expression vector. Using these expression plasmids, we developed a two-plasmid genetic complementation assay to monitor the packaging of FIV unspliced sub-genomic RNA (Figure 1B). Briefly, the assay was based on the premise that the FIV sub-genomic transfer vector RNA (MB87; Figure 1A; [36]) contains the already-established packaging determinants of FIV [37,38,43] which could be recognized by the FIV full-length Pr50^Gag^ protein, facilitating its encapsidation/packaging in the nascently forming virus particles. Towards this end, both the His_6_-tag (+) and His_6_-tag (−) FIV Pr50^Gag^ expression plasmids (AD2 and AD3, respectively) were individually co-transfected along with the FIV transfer vector, MB87, in 293T cells (Figure 1B). Electron micrographs taken from different fields of the transfected 293T cells revealed VLP production both in the presence and absence of the His_6_-tag. As shown in Figure 2, an electron dense protein layer was observed underlying the plasma membrane from which spherical shaped VLPs could be seen budding when FIV Pr50^Gag^ expression plasmids with His_6_-tag (AD2) (Figure 2A–D) or without His_6_-tag (AD3) (Figure 2E–H) were transfected in 293T cells. In mock experimental controls (cells that were transfected with the void expression plasmid pcDNA3), as expected, no budding of VLPs could be observed (Figure 2I–J). The size of the majority of VLPs produced was calculated to be ~100–120 nanometers (nm) in diameter (Figure 2), which is slightly smaller than the mature FIV particles (125–150 nm) and consistent with the release of immature and spherical FIV particles from eukaryotic cells, as reported earlier [65,66]. These results demonstrate that we were successful in expressing the recombinant full-length FIV Pr50^Gag^ protein both with and without His_6_-tag in eukaryotic cells, and the expressed proteins could make VLPs. Therefore, cloning His_6_-tag at the C-terminus of Gag did not interfere with the expression of the recombinant full-length FIV Pr50^Gag^ or the production of VLPs.

Next, supernatants from the transfected cultures were collected, and the immature viral particles were pelleted by ultracentrifugation. RNA from immature viral particles was isolated and subjected to RT-PCR to monitor the ability of the His (+) and His (−) Pr50^Gag^ proteins to package FIV sub-genomic RNA. Towards this end, RNA preparations were DNase-treated and cDNAs prepared, as described in Materials and Methods. The use of conventional RT-PCR revealed that immature viral particles formed by both His (+) and His (−) Pr50^Gag^ proteins could package RNA from the sub-genomic transfer vector, MB87, in an efficient manner (Figure 1C; upper panel). To assess the relative ability of the His (+) and His (−) Gag proteins to encapsidate FIV subgenomic RNA, an in-house SYBR Green-based qPCR assay was developed in which β-actin was used as an endogenous control, as described in Materials and Methods. The relative transfer vector (MB87) RNA packaging efficiency (RPE) by the His (+) and His (−) Gag proteins was determined by calculating the ratio of the transfer vector (MB87) RNA packaged in the VLPs to the transfer vector (MB87) RNA being expressed in the cytoplasm. Using this assay, essentially a similar ability of the two proteins to package MB87 RNA was observed (Figure 1C; lower panel). This observation confirmed that cloning the His_6_-tag at the C-terminus did not interfere with the expression of the recombinant full-length FIV Pr50^Gag^ or production of VLPs, nor did it negatively affect its ability to package FIV unspliced sub-genomic RNA. This is also consistent with the earlier observations made in the case of MPMV and MMTV, where VLPs produced in eukaryotic cells by full-length Gag both in the presence or absence of His_6_-tag were able to package nearly equal amounts of their respective gRNAs [14,15]. Finally, a close corroboration in the functional capacities of recombinant full-length FIV Pr50^Gag^ proteins (both with and without His_6_-tag) was further supported by the fact that when sequences for AD2 (with His_6_-tag) and AD3 (without His_6_-tag) were investigated using the online ExPASy-Compute pI/Mw tool to calculate the theoretical isoelectric point (pI) of the proteins, predicting almost no difference (0.07) in the expressed recombinant proteins (AD2 with His_6_-tag: pI: 8.92 versus AD3 without His_6_-tag: pI: 8.99).

### 3.2. Expression of Full-Length Recombinant FIV Pr50^Gag^-His_6_-Tagged Fusion Protein in Prokaryotic Cells

**Figure 3:**
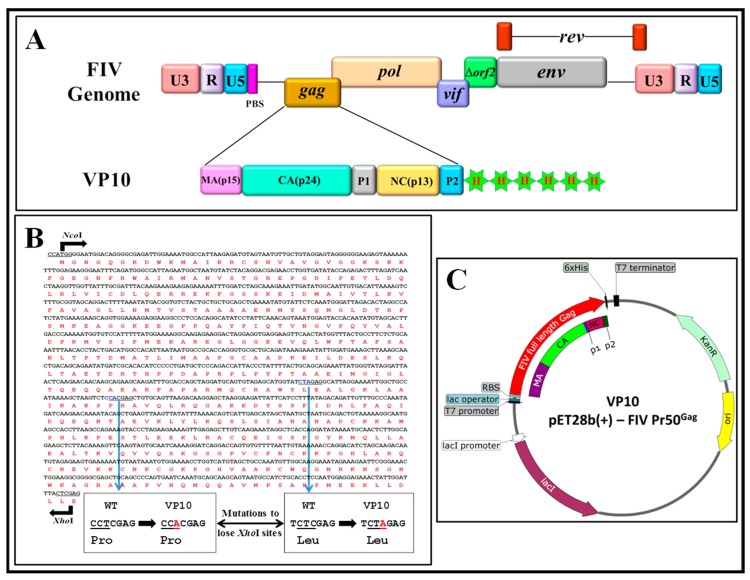
Graphical representation of cloning of the FIV genome and full-length Pr50^Gag^-His_6_-tag bacterial expression plasmid. (**A**) Schematic representation of the FIV genome and VP10 FIV full-length Pr50^Gag^ bacterial expression plasmid showing Gag precursor domains and His_6_-tag. (**B**) FIV full-length Pr50^Gag^ nucleotide and amino acid sequences with mutations introduced (shown as insets) to lose two *Xho*I sites for cloning purposes without changing the amino acid sequence. (**C**) Schematic representation of VP10- FIV full-length Pr50^Gag^ after cloning Gag sequences in the modified pET28b (+) vector in which Pr50^Gag^ is expressed from the bacteriophage T7 promoter.

Having established the expression and functional characterization of the recombinant FIV Pr50^Gag^-His_6_-tagged fusion protein in eukaryotic cells, we next embarked on expressing and purifying this protein from *E. coli*. Towards this end, the FIV full-length Gag was cloned into the prokaryotic expression plasmid, pET28b(+), to generate VP10 (Figure 3), as described in Materials and Methods. Such a cloning strategy should generate a fusion protein expressing the full-length FIV Pr50^Gag^ containing a LEHHHHHH tag at the C-terminus (Pr50^Gag^-His_6_-tag), with a predicted molecular weight of 50.3 kDa. The transformation of VP10 plasmid DNA into BL21 (DE3) bacterial cells allowed high level expression of the recombinant protein when induced by IPTG.

**Figure 4:**
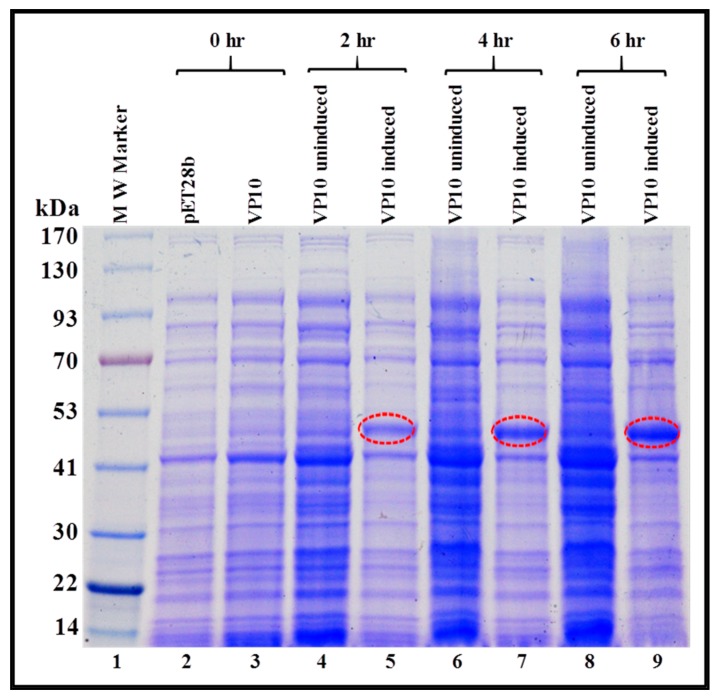
Expression of a recombinant FIV full-length Pr50^Gag^-His_6_-tag fusion protein in *Escherichia coli* (*E. coli*) lysates. BL21(DE3) cells transformed with VP10 were grown overnight at 37 °C, and then induced with IPTG and further cultured for varying time points at 37 °C. Total bacterial cell lysates were prepared from uninduced and IPTG-induced cultures and analyzed by SDS-PAGE. Coomassie Brilliant Blue-stained SDS-PAGE confirms that full length Pr50^Gag^-His_6_-tag fusion protein is expressed efficiently from VP10.

As a first step, we monitored the expression of VP10-transformed BL21 (DE3) cultures in total bacterial lysates at 0, 2, 4, and 6 hours at 37 °C. Briefly, total protein lysates were prepared for each time point from both IPTG-induced as well as uninduced cultures and examined by SDS-PAGE. As shown in Figure 4, a discrete band of approximately 50 kDa could be detected exclusively in cultures from 2, 4, and 6 h post IPTG induction (lanes 5, 7, and 9), and not in cultures at 0 hour (lane 3), uninduced cultures (lanes 4, 6, and 8), or in cultures transformed with only the expression plasmid without any FIV Pr50^Gag^ sequences (pET28b alone; lane 2). These observations demonstrate that a recombinant full-length FIV Pr50^Gag^ protein could be expressed in bacteria. However, they did not show whether the recombinant protein was present in the soluble fraction from which it could be purified or if it confined to inclusion bodies, as has been previously reported in the case of MPMV [67].

### 3.3. FIV Pr50^Gag^-His_6_-Tagged Fusion Protein Is Expressed in the Soluble Fraction in Bacteria

**Figure 5:**
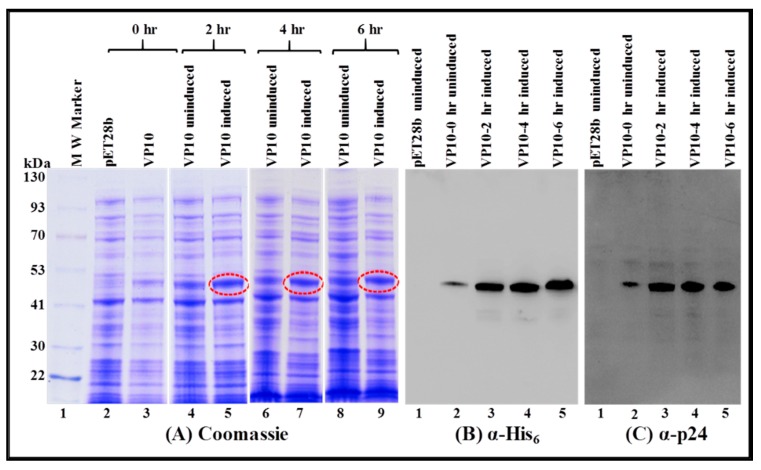
Expression of the recombinant FIV full-length Pr50^Gag^-His_6_-tag fusion protein in a soluble fraction of *Escherichia coli* (*E. coli*). Bacterial cells transformed with VP10 were grown at 37 °C, then induced with IPTG and further cultured for varying time points at 37 °C. Soluble fractions prepared from uninduced and IPTG-induced cultures were electrophoresed on SDS-polyacrylamide gel. (**A**) Coomassie Brilliant Blue-stained gel confirmed that the full length Pr50^Gag^-His_6_-tag fusion protein expressed efficiently from VP10 is present in the soluble fraction. Western blot analyses on the same soluble fractions from bacteria containing the Pr50^Gag^-His_6_-tag fusion protein were conducted using α-His_6_ (**B**) and α-FIV p24 (**C**) monoclonal antibodies, respectively, which further confirmed the presence of the fusion protein in the soluble fractions.

**Figure 6:**
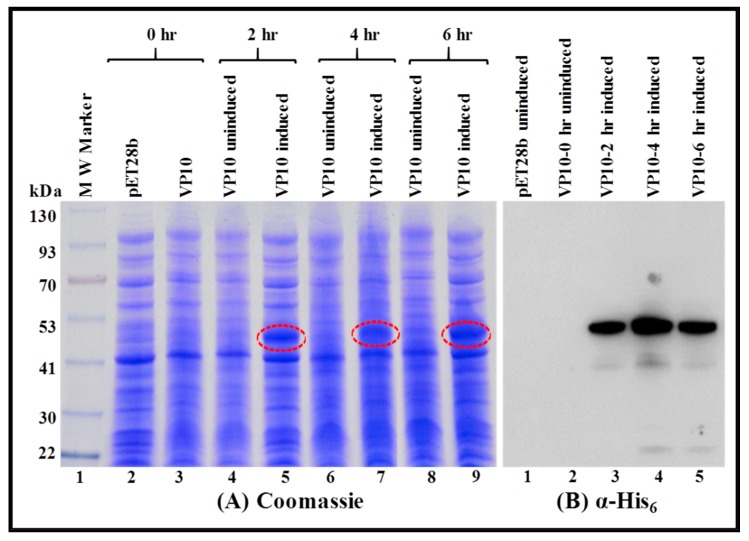
Sub-optimal expression of the recombinant FIV full-length Pr50^Gag^-His_6_-tag fusion protein in soluble fraction of *Escherichia coli* (*E. coli*). Bacterial cells transformed with VP10 were grown at 37 °C and induced with IPTG. This was followed by growing the cultures sub-optimally for varying time points at 28 °C in the presence of 1% glucose. (**A**) Coomassie Brilliant Blue-stained electrophoresed gel confirmed expression of a full length Pr50^Gag^-His_6_-tag fusion protein from VP10 in the soluble fraction. (**B**) Western blot analysis on the same soluble fraction from bacteria containing Pr50^Gag^-His_6_-tag fusion protein using α-His_6_ monoclonal antibody.

To establish that the recombinant full-length FIV Pr50^Gag^-His_6_-tag protein was expressed in soluble fractions, an overnight bacterial culture of VP10-transformed in BL21(DE3) bacterial cells was induced with IPTG and allowed to grow at 37 °C for 2, 4, and 6 h. Cells harvested at various time points were pelleted and processed as described in the Materials and Methods section. Cell debris and/or inclusion bodies, if any, and other insoluble material, were removed by centrifugation, and the soluble fraction of the full-length FIV Pr50^Gag^-His_6_-tag fusion protein obtained from different time points was analyzed by SDS-PAGE and immunoblotting. SDS-PAGE analysis following Coomassie Brilliant Blue staining revealed a characteristic band of ~50 kDa consistent with the anticipated size of the recombinant FIV Pr50^Gag^-His_6_-tag protein from IPTG-induced cultures (Figure 5A; lanes 5, 7, and 9). Next, we established the uniqueness and recombinant nature (Pr50^Gag^-His_6_-tag fusion protein) of the bands observed on Coomassie Brilliant Blue staining by western blotting using HRP-conjugated α-His_6_ as well as FIV α-p24 monoclonal antibodies (Figure 5B,C; lanes 3, 4, and 5, respectively). Immunoblotting employing both α-His_6_ as well as FIV α-p24 monoclonal antibodies of soluble fractions from VP10 uninduced cultures exhibited some expression of FIV Pr50^Gag^-His_6_-tag fusion protein (Figure 5B; lane 2 and Figure 5C; lane 2). Such low-level expression in the uninduced culture could possibly be due to the leaky nature of the T7 promoter of the expression plasmid, a phenomenon that has been reported earlier [68]. In order to inhibit promoter leakiness, we opted to express the Pr50^Gag^-His_6_-tag fusion protein from VP10 following IPTG induction using sub-optimal conditions, such as low temperature (28 °C) instead of 37 °C and growth in the presence of glucose, as described previously [68]. Such a strategy has been successful in suppressing the leaky nature of the T7 promoter of the expression plasmid during MMTV Pr77^Gag^ expression [15]. Consistent with our expectations on Coomassie Brilliant Blue staining, the recombinant Pr50^Gag^-His_6_-tagged fusion protein was observed exclusively in the IPTG-induced cultures (Figure 6A; lanes 5, 7, and 9) and not in the uninduced cultures (Figure 6A; lanes 4, 6, and 8). Immunoblotting of soluble fractions from VP10 using α-His_6_ monoclonal antibody also confirmed the lack of expression of the recombinant full-length FIV Pr50^Gag^-His_6_-tag protein from uninduced cultures (Figure 6B; lane 2). These results clearly demonstrate that a FIV Pr50^Gag^-His_6_-tag protein can be expressed in the soluble fractions of bacterial lysates employing sub-optimal culturing conditions, with the maximal expression at 4 hours (Figure 6A; lane 7 and Figure 6B; lane 4). Despite the fact that the recombinant FIV full-length Pr50^Gag^-His_6_-tag expression could be observed between 2 and 6 hours following induction by IPTG (Figure 6A; lanes 5, 7, and 9 and Figure 6B; lanes 3, 4, and 5), for further large scale purification, we decided to express the protein sub-optimally only (28 °C) and for 4 h post IPTG induction to circumvent any possible accumulation and/or confinement, as has been reported in the case of the MPMV Gag polyprotein by culturing bacteria at 37 °C for extended periods of time [67].

### 3.4. Purification of the Recombinant Full-Length Pr50^Gag^-His_6_-Tagged Fusion Protein from Soluble Fraction by Immobilized Metal Affinity Chromatography (IMAC)

**Figure 7:**
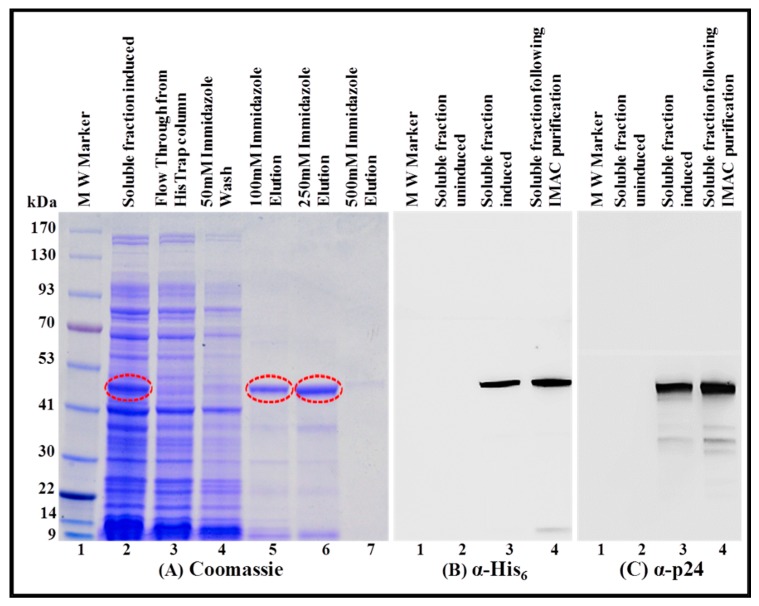
Purification of the FIV recombinant Pr50^Gag^-His_6_-tag protein from soluble fractions by immobilized metal affinity chromatography (IMAC) and its western blot analysis. (**A**) Coomassie Brilliant Blue-stained SDS-polyacrylamide gel on which bacterial soluble fractions containing FIV Pr50^Gag^-His_6_-tag fusion protein was electrophoresed before and after IMAC purification using different concentrations of immidazole. Western blot analyses on the same soluble fractions (before and after IMAC purification) using α-His_6_ (**B**) and FIV α-p24 (**C**) monoclonal antibodies, respectively, using equal amounts of protein.

Following the confirmation that the FIV recombinant full-length Pr50^Gag^-His_6_-tagged fusion protein is indeed present in the soluble fraction, we embarked on purification of the protein by IMAC, as has recently been accomplished for the HIV-1, MPMV, and MMTV full-length Gag proteins ([13,14,15]; see Materials and Methods for details). During IMAC purification, we made sure to use non-denaturing buffer conditions (especially 1.0 M NaCl concentration) that enable the binding of the protein to the column and at the same time avoid protein aggregation and precipitation. Briefly, soluble fraction from the bacterial lysates were applied to the Ni-immobilized HisTrap fast flow (FF) column, which allowed the protein to bind to the column. Consequently, no protein could be visualized in the flow through from the HisTrap column or in the 50 mM immidazole wash (Figure 7A; lanes 2, 3, and 4). Next, the HisTrap-column bound protein was eluted using different concentrations of imidazole (100 mM, 250 mM, and 500 mM) with the maximum protein eluting at 250 mM imidazole (Figure 7A; lanes 5, 6, and 7). IMAC purification was able to get rid of most of the bacterial proteins present in the soluble fraction (Figure 7A; compare lane 2 with lane 6). Following IMAC purification, the purity of the recombinant FIV Pr50^Gag^-His_6_-tag fusion protein was further established by immunoblotting using HRP-conjugated α-His_6_ (Figure 7B; lane 4) as well as FIV α-p24 monoclonal antibodies (Figure 7C; lane 4). Taken together, these observations validate that the recombinant FIV full-length Pr50^Gag^ protein is indeed fused in a frame with His_6_-tag, which facilitated its retention on the HisTrap column and its elution into a purer form (Figure 7A; compare lane 2 with lane 6; Figure 7B; lane 4; Figure 7C lane 4). The IMAC-purified Pr50^Gag^-His_6_-tagged fusion protein, upon immunoblotting with the FIV α-p24 antibody, still revealed additional faint bands (Figure 7B; lane 4 and Figure 7C; lane 4), which could be attributed to the possible degradation of the recombinant protein.

### 3.5. Further Purification of the IMAC-Purified Pr50^Gag^-His_6_-Tag Protein by Gel Filtration/Size Exclusion Chromatography

After having confirmed the recombinant nature of the Pr50^Gag^-His_6_-tagged protein eluted following IMAC purification, the protein was concentrated using an Amicon^®^ Ultra 15 centrifugal device (30 kDa cut-off membrane). Next, the concentrated protein was further purified by gel filtration/size exclusion chromatography under non-denaturing conditions using a Superdex 200 10/300 GL column, as described previously [14,15]. As in the case of IMAC purification, any possible protein aggregation and precipitation was avoided by using a high salt concentration (1.0 M NaCl) in the gel filtration buffer. On the basis of absorbance at 280 nm, which suggested elution of the purified recombinant protein, 500 µL fractions were collected and analyzed by SDS-PAGE and immunoblotting.

**Figure 8:**
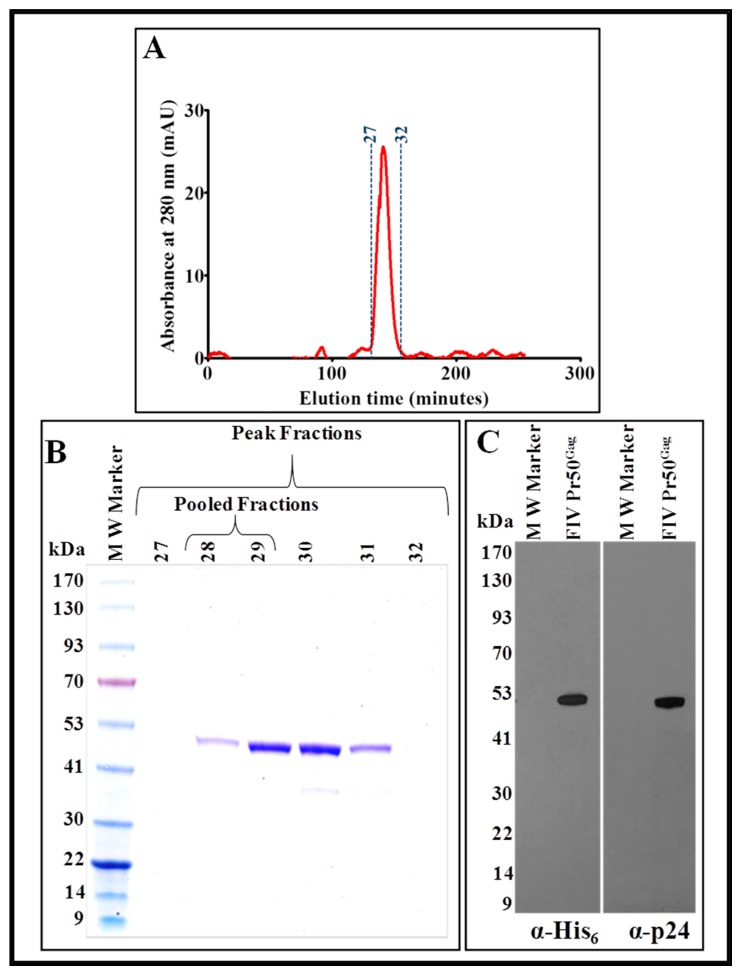
Size exclusion chromatography and western blot analysis of the IMAC-purified recombinant Pr50^Gag^-His_6_-tag protein. (**A**) Chromatogram showing a distinct peak (between fractions 27 and 32) of the purified recombinant full-length FIV Pr50^Gag^-His_6_-tag fusion protein. (**B**) Coomassie Brilliant Blue-stained SDS-PAGE following electrophoresis of fractions 27–32, which reveals the presence of the purest form of the recombinant full-length FIV Pr50^Gag^-His_6_-tag fusion protein in fractions 28 and 29. (**C**) Western blot analyses on the pooled fractions (fractions 28 and 29) of the purified protein using α-His_6_ and FIV α-p24 monoclonal antibodies, respectively, using equal amounts of protein.

As shown in Figure 8A, fractions 27–32 of the eluted protein corresponded to a distinct peak at A_280_. Analysis of these fractions by SDS-PAGE and Coomassie Brilliant Blue staining of the gel revealed that the distinct peak consisted of variable amounts of pure FIV Pr50^Gag^-His_6_-tag fusion protein (Figure 8B). These fractions (28–29) containing the purest form of the protein (without any bands other than of Pr50^Gag^) were combined and again concentrated using Amicon® Ultra 15 centrifugal columns (30 kDa cut-off membrane) to bring the protein to a final concentration of ~2 mg/mL.

The concentrated protein was further analyzed by immunoblotting using HRP-conjugated α-His_6_ as well as FIV α-p24 monoclonal antibodies. The results depicted in Figure 8C corroborated well with the SDS-PAGE analysis (Figure 8B), demonstrating that the pooled protein fractions contained a pure FIV Pr50^Gag^-His_6_-tag fusion protein. Finally, we estimated the purity of the purified protein by measuring the A_260_/A_280_ ratio spectrophotometrically, which gave a value of 0.62, suggesting that the protein purity was greater than 95% and contained only an inconsequential amount of nucleic acid impurities, if any. In terms of yield, a one-and-a-half-liter bacterial culture yielded ~16 mg of protein following IMAC purification, from which two milligrams of the purest form of recombinant Pr50^Gag^-His_6_-tag fusion protein could be recovered following gel filtration/size exclusion chromatography.

### 3.6. The Recombinant Full-Length Pr50^Gag^-His_6_-Tagged Fusion Protein Can Assemble In Vitro to Form Virus-Like Particles (VLPs)

The ability of retroviral Gag proteins to self-assemble in vitro and form VLPs for lentiviruses such as HIV-1 and FIV, and other retroviruses, such as MPMV and MMTV, has been reported earlier [13,14,15,59,60,69]. Therefore, as a logical next step, to further validate the purity and biological activity of the recombinant full-length FIV Pr50^Gag^-His_6_-tag fusion protein expressed in prokaryotic cells, we monitored the ability of this protein to form VLPs by assembling itself in vitro. Since the presence of nucleic acids has been found to be necessary for in vitro assembly and VLP formation [13,59,60,69], these experiments were carried out in the presence of 4% *w*/*w* yeast tRNA, as described previously [13,14,15]. To circumvent the aggregation of the purified recombinant FIV Pr50^Gag^-His_6_-tag fusion protein, the protein was mixed with yeast tRNA in a high salt buffer (1 M NaCl) and dialyzed against a buffer with relevant physiological salt concentration. As an ancillary control, the purified recombinant FIV Pr50^Gag^-His_6_-tag fusion protein without yeast tRNA was also dialyzed in a similar fashion. Dialysis was carried out overnight at 4 °C, following which samples were collected individually from the dialysis cassette, and 10 µL of the 500 µL assembly reaction mixture was spotted on formvar carbon coated grids, negatively stained, and visualized by TEM.

**Figure 9:**
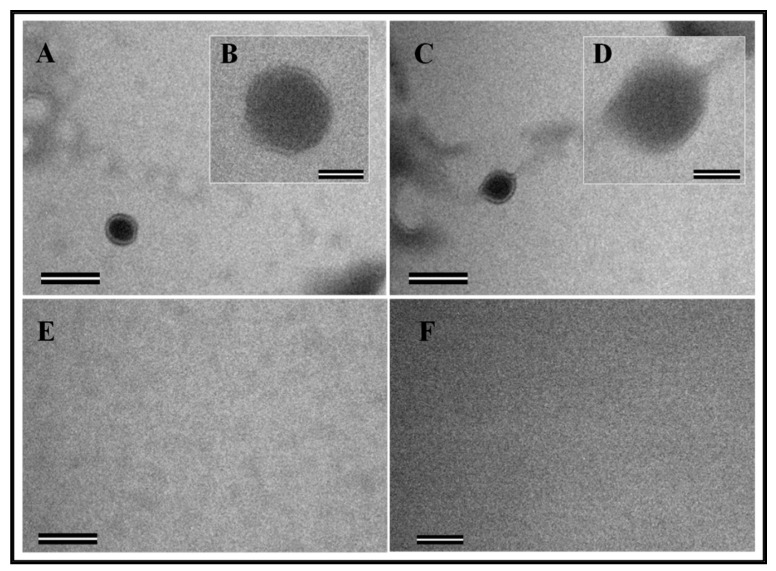
The ability of the purified recombinant Pr50^Gag^-His_6_-tag fusion protein to assemble in vitro to form virus-like particles (VLPs). (**A**–**D**) Transmission electron micrographs showing the morphology of the FIV Gag particles assembled in vitro in the presence of yeast tRNA. (**B**,**D**) Electron micrographs taken at higher magnifications are shown as insets. (**E**,**F**) Electron micrographs of negative control samples composed of an in vitro assembly buffer and purified protein in the absence of yeast tRNA. Scale bars represent 200 nm (43,000× magnification) for the wide field pictures and 50 nm (135,000× magnification) for the insets, respectively.

The electron micrographs of the structures assembled in vitro by the recombinant FIV Pr50^Gag^-His_6_-tag fusion protein were taken from different fields. As expected, the formation of VLPs in the form of compact electron-dense spherical rings of approximately 89 nm in size confirmed the in vitro assembly process when the recombinant FIV Pr50^Gag^-His_6_-tag fusion protein was mixed with yeast tRNA (Figure 9A–D). On the other hand, as reported earlier [59], the recombinant FIV Pr50^Gag^-His_6_-tag fusion protein alone (without yeast tRNA) could not be assembled in vitro to form VLPs (Figure 9E–F). However, the size (~89 nm) of the in vitro assembled FIV Pr50^Gag^-His_6_-tag particles observed in this study was comparatively larger than the earlier reported size (~33 nm) of FIV Gag in vitro assembled particles [59]. However, both these observations are consistent with other published reports, where the smaller size of the in vitro assembled FIV Pr50^Gag^-His_6_-tag particles is more similar to observations made in a number of other retroviruses, including HIV-1, showing a smaller size of the in vitro assembled particles when compared to the Gag particles assembled in vivo [13,59,60,65,69]. Taken together, these results demonstrate that the purified recombinant FIV Pr50^Gag^-His_6_-tag fusion protein has the intrinsic ability to assemble in vitro to form VLPs when incubated with nucleic acids.

## 4. Conclusions

In this study, we successfully expressed and purified a full-length recombinant FIV Pr50^Gag^ protein with a C- terminus His_6_-tag in a bacterial expression system. The resultant purified recombinant protein had the ability to assemble in vitro to form VLPs. In vivo VLPs formed by the recombinant FIV Pr50^Gag^ with and without a His_6_-tag showed the ability to specifically recognize and package FIV sub-genomic RNA at equal levels. The ability to express and purify a full-length recombinant Pr50^Gag^-His_6_-tag fusion protein will further improve our understanding of the molecular mechanisms underlying various steps in retroviral replication, such as RNA dimerization, packaging, and virus assembly. In vitro binding assays using the purified full-length recombinant Pr50^Gag^-His_6_-tag fusion protein could further delineate the mechanism of Gag-RNA interactions during FIV gRNA packaging. Furthermore, the availability of a purified biologically active full-length Pr50^Gag^-His_6_-tag fusion protein could allow cryo-EM studies to understand the structural interactions involved in the complex process of Gag multimerization and assembly.
